# Telomeres open a window on stem cell division

**DOI:** 10.7554/eLife.12481

**Published:** 2016-01-25

**Authors:** Ignacio A Rodriguez-Brenes, Dominik Wodarz

**Affiliations:** 1 Department of Mathematics and the Department of Ecology and Evolutionary Biology, University of California Irvine, Irvine, United States; 2 Department of Ecology and Evolutionary Biology and the Department of Mathematics, University of California Irvine, Irvine, United Statesdwodarz@uci.edu

**Keywords:** telomere length distribution, stem cells, mathematical modelling, hematopoiesis, self renewal, personalised medicine, Human

## Abstract

Measuring the length distribution of telomeres can reveal information about biological processes that are otherwise difficult to analyze experimentally.

**Related research article** Werner B, Beier F, Hummel S, Balabanov S, Lassay L, Orlikowsky T, Dingli D, Brümmendorf TH, Traulsenz A. 2015. Reconstructing the *in vivo* dynamics of hematopoietic stem cells from telomere length distributions. *eLife* **4**:e08687. doi: 10.7554/eLife.08687

**Image** Telomere length was measured in a range of blood tissue samples

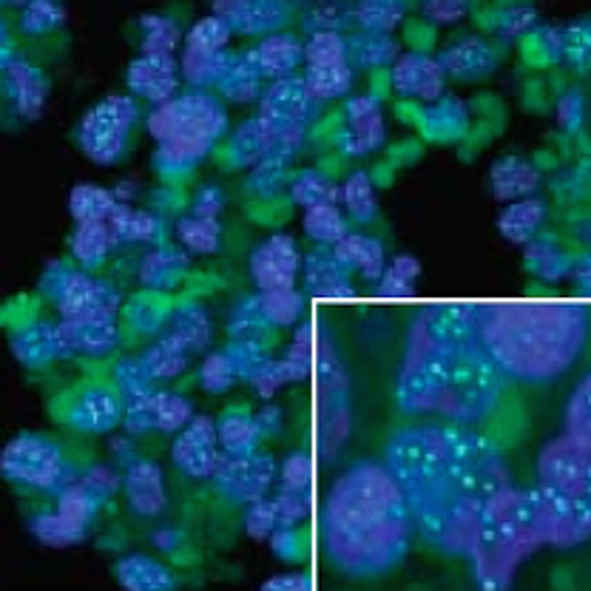

Stem cells are undifferentiated cells that can develop into more specialized cells. There are two kinds of stem cells: embryonic stem cells and adult stem cells. Embryonic stem cells are active during early development and give rise to all the different cell types in the body. Adult stem cells are specific to each tissue and give rise to all the specialized cells in a particular tissue or organ. When a stem cell divides, each new cell has the potential to either remain a stem cell or differentiate into a more specialized type of cell (Figure 1A). However, it can be difficult to analyze these division patterns in humans. Now, in eLife, Benjamin Werner, Fabian Beier, Arne Traulsen and colleagues have used a mathematical model to reconstruct the dynamics of blood stem cells from measurements of telomere length (Werner et al., 2015).

**Figure 1.**
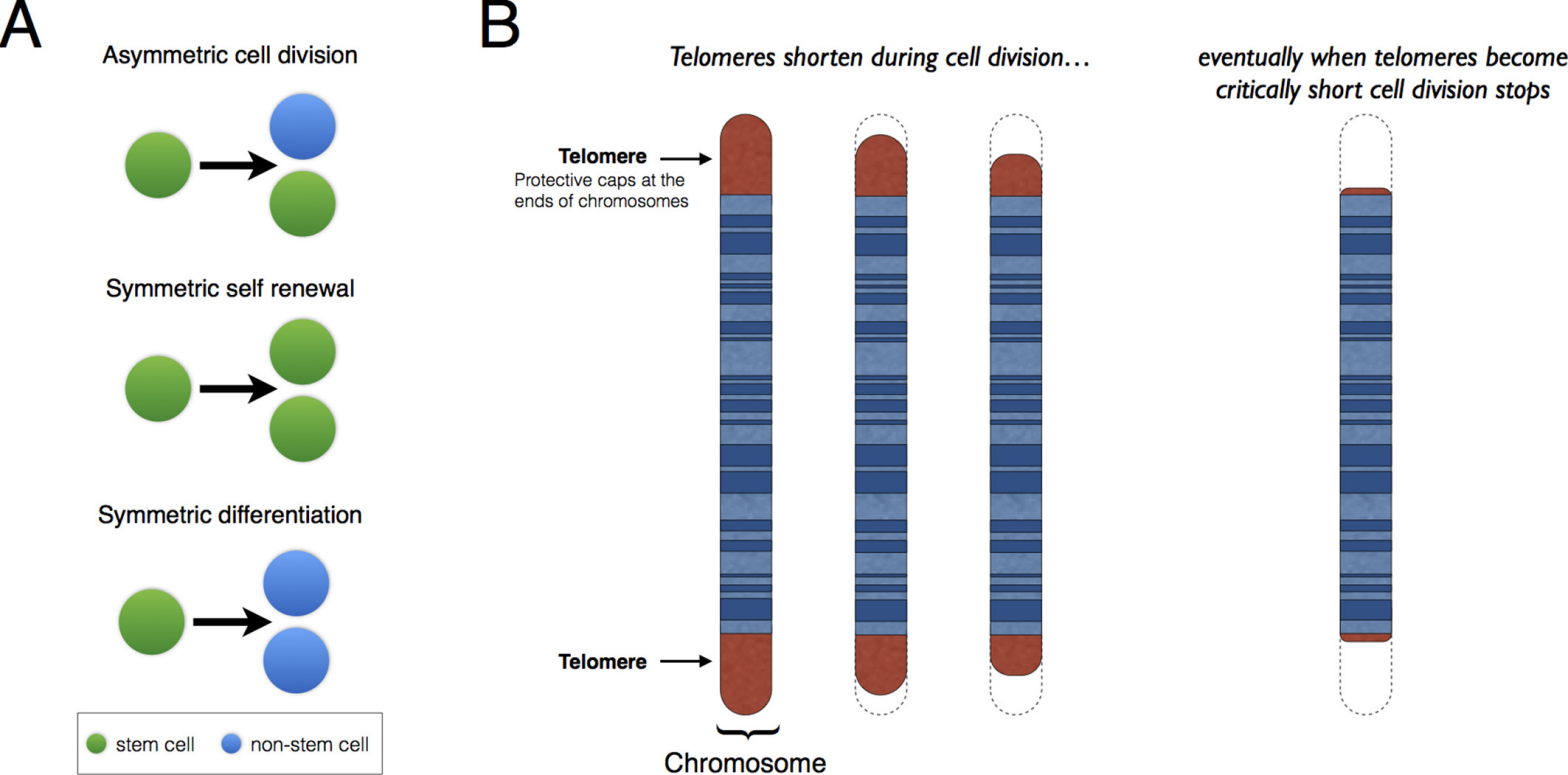
Patterns of stem cell division and the protective role of telomeres. (**A**) Stem cells can divide asymmetrically to produce a new stem cell and a non-stem cell that can differentiate to replace other types of cells that are lost from the tissue because of cell death (top). Stem cells can also divide symmetrically, producing two daughter stem cells (symmetric self-renewal, middle) or two non-stem cells (symmetric differentiation, bottom). (**B**) Telomeres are the regions of DNA that cap and protect the ends of linear chromosomes. Each time a cell divides the telomeres get shorter. When telomeres become too short, cell division stops.

Telomeres are lengths of DNA that cap both ends of linear chromosomes (Figure 1B), and they protect the chromosomes by preventing their natural ends from being interpreted as breaks in the DNA. During cell division, the enzymes that duplicate DNA cannot copy the very ends of chromosomes; this ‘end-replication problem’ is part of the reason why the telomeres get shorter each time a cell divides (Martinez and Blasco, 2015). When telomeres become very short, they lose their protective properties and cell division stops. This process is known as ‘replicative senescence’ and is correlated with aging: put simply, telomeres get shorter as people get older.

Replicative senescence is believed to have evolved as a means to curb excessive cell division, which is a hallmark of cancer. However, human cancers find ways to bypass this process, typically by expressing an enzyme called telomerase that acts to lengthen the telomeres. Telomerase is highly active in embryonic stem cells, but it is not expressed in most normal cells.

Werner, Beier, Traulsen and colleagues – who are based at the Max Planck Institute for Evolutionary Biology, RWTH Aachen University Hospital, University Hospital Zürich and the Mayo Clinic – measured the average telomere lengths from blood samples taken from 356 individuals aged between 0 and 85 years old. Two alternative models of stem cell dynamics were then analyzed. The first model considered that the stem cells only divide asymmetrically, producing one stem cell and one non-stem cell. The second model included both asymmetric cell division and symmetric self-renewal (where a stem cell divides to form two daughter stem cells; Figure 1A). Werner, Beier et al. found that the first model predicted a linear relationship between average telomere length and the donor’s age, whereas the second model predicted a nonlinear decrease in telomere length. The data strongly favored the second model.

The findings suggest that symmetric self-renewal is more frequent during adolescence. Since symmetric self-renewal could promote the accumulation of mutations (Tomasetti and Vogelstein, 2015), this has implications for understanding how cancer emerges. A previous theoretical study argued that the high number of cell divisions that occur during fetal development puts us at risk of acquiring mutations even before birth (Frank and Nowak, 2003). The new results extend this argument into childhood and adolescence. That is, before adulthood is reached, there is possibly a relatively high risk of acquiring mutations that may predispose an individual to cancer – even if the onset of cancer typically occurs much later in life.

An important question that arises from this study concerns the exact nature of the cell divisions that ensure tissue maintenance in adulthood. In the model of Werner, Beier et al., tissues are maintained in adulthood through asymmetric cell divisions. However, as they point out, this model cannot be mathematically distinguished from an alternative mechanism that relies on a mixture of symmetric self-renewal and symmetric differentiation (i.e., when the stem cell divides to produce two non-stem cells). This is because tissues can also be maintained if the probabilities of symmetric self-renewal and differentiation are balanced and controlled through feedback loops (Lander et al., 2009); this latter model is supported by stem cell data from both humans and other animals (see Shahryiari and Komarova, 2013 for references). Some mathematical models suggest that the prevalence of mutations depends on the division patterns, so it may be possible to distinguish between them mathematically (Shahryiari and Komarova, 2013).

Using telomere length as an indicator of biological processes is not a new idea. Telomere length has previously been singled out as a marker to identify adult stem cells and their location in the body (Flores et al., 2008). From a modeling perspective, the length of telomeres has also been proposed as a signal to assess the risk posed by pre-cancerous mutations in healthy individuals (Rodriguez-Brenes et al., 2014). Telomere length might also help predict the success rate of cancer therapies, and studies of telomerase inhibitors predict better outcomes for patients with short telomere length (Chiappori et al., 2015). The work by Werner, Beier et al. adds an important contribution in this context, allowing us to use telomere lengths to gain insights into cell division patterns that occur in vivo.
